# Placental DNA methylation as a proxy for fetal neurodevelopment and sex-specific associations with in utero particulate air pollution

**DOI:** 10.1186/2049-3258-73-S1-P35

**Published:** 2015-09-17

**Authors:** Nelly Saenen, Bram Janssen, Karen Vrijens, Harry Roels, Wim Vanden Berghe, Wilfried Gyselaers, Charlotte Vanpoucke, Patrick De Boever, Tim Nawrot

**Affiliations:** 1 Hasselt University, Diepenbeek, Belgium; 2 Department of Biomedical Sciences, PPES Epigenetic Signaling Lab, University of Antwerp, Antwerp, Belgium; 3 Department of Obstetrics, East-Limburg Hospital, Genk, Belgium; 4 Belgian Interregional Environment Agency, Brussels, Belgium; 5 Flemish Institute for Technological Research (VITO), Mol, Belgium

## Background and aims

Exposure to particulate matter (PM) air pollution during pregnancy may affect human fetal development. Epigenetic mechanisms are believed to play an essential role in the developmental changes during early life. Within the ENVIR*ON*AGE birth cohort, we investigated whether *in utero* exposure to PM is associated with differences in placental DNA methylation of genes involved in early neurodevelopment, i.e., Brain-Derived Neurotrophic Factor (*BDNF*), Leptin (*LEP*) and 5-Hydroxytryptamine (serotonin) receptor 2A (*HTR2A*).

## Methods

Using highly quantitative bisulfite-PCR pyrosequencing, DNA promoter methylation was assessed in placental tissue of 385 newborns from the ENVIR*ON*AGE birth cohort. Daily PM_2.5_ exposure levels were estimated for each participant's home address using a spatiotemporal interpolation model in combination with a dispersion model. We fitted mixed-effect models, stratified for newborn's sex, to evaluate the associations between DNA promoter methylation of the selected genes and PM_2.5_ exposure during pregnancy.

## Results

Methylation of placental *BDNF* in male infants rose by 0.46% (*p* = 0.02) for an interquartile range (IQR) increment in PM_2.5_ during the second trimester of pregnancy. For placental *HTR2A*, methylation in male infants rose by 4.8% (*p* = 0.02) for an IQR increment in first trimester PM_2.5_ exposure. These associations were independent of maternal age, maternal education, maternal smoking status, gestational age, CpG site, first trimester temperature, and season at birth. No associations were observed between PM_2.5_ exposure and placental *LEP methylation.* In girls no significant associations were noted.

## Conclusions

Placental promoter methylation of *BDNF* and *HTR2A*, two genes implicated in early neurodevelopmental trajectories, are influenced by *in utero* exposure to PM_2.5_ in a sex-specific way. Future studies should elucidate the significance of the sex-specific PM_2.5_ impact on placental promoter methylation with respect to neurodevelopment later in life.

